# Neurocognitive outcomes following glioma diagnosis and treatment: A systematic review

**DOI:** 10.1016/j.bas.2026.106663

**Published:** 2026-09-04

**Authors:** Maria D. Karagianni, Christina Arvaniti, Evie-Paraskevi Fountas, Antonios Benos, Alexandros Brotis, Konstantinos N. Fountas

**Affiliations:** a Department of Neurosurgery, University General Hospital of Larissa, Biopolis, Larissa, 41110, Greece; b The Chicago School of Professional Psychology, Business Psychology Department, Chicago, IL, 60610, USA; c Faculty of Medicine, School of Health Sciences, University of Thessaly, Biopolis, Larissa, 41110, Greece

**Keywords:** Behavioral disorders, Cognition disorders, Glioma, Neuropsychological tests, Quality of life, Treatment outcome

## Abstract

**Introduction:**

Gliomas are the most common primary malignant brain tumors in adults and are often accompanied by cognitive disturbances, which profoundly affect daily functioning and quality of life. Although survival and neurological deficits are routinely reported, neurocognitive outcomes remain insufficiently integrated into clinical decision-making.

**Research question:**

This review seeks to describe the patterns and progression of neurocognitive dysfunction in adult glioma patients and to clarify how tumor characteristics, patient-related factors, and treatment strategies influence cognitive outcomes. Additionally, we explore whether cognitive function may serve as a predictor of clinical outcome.

**Material and methods:**

A systematic literature review was performed following PRISMA guidelines. PubMed, Scopus, and Web of Science were searched from inception to August 2025. Studies involving adult glioma patients assessed with standardized neurocognitive instruments and reporting cognitive outcomes in relation to tumor features or treatment modalities were included. Study quality was evaluated using established risk-of-bias tools.

**Results:**

Twenty-five studies met our inclusion criteria. Cognitive impairment was frequently present at diagnosis, particularly in patients with tumors affecting eloquent brain regions. Surgical treatment was commonly associated with short-term cognitive decline, followed by stabilization or improvement in many patients over time. The effects of chemotherapy and radiotherapy on cognition were inconsistent. Tumor location, laterality, and histopathological grade emerged as key factors influencing cognitive trajectories. Preserved cognitive function was also associated with better quality of life and overall survival.

**Discussion and conclusion:**

Neurocognitive dysfunction in glioma patients is common, heterogeneous, and shaped by multiple interacting factors. Routine, standardized cognitive assessment and longitudinal follow-up are essential to better capture these changes and to incorporate cognitive outcomes into individualized glioma care.

## Highlights

• Cognitive impairment is frequent at diagnosis and varies by tumor location and grade.
• Surgery causes transient decline, with later stabilization or improvement in many cases.
• Awake mapping supports cognitive preservation in eloquent-area glioma surgery.
• Cognitive effects of chemotherapy and radiotherapy remain inconsistent across studies.
• Preserved cognition correlates with improved quality of life and survival outcomes.

## 1 Introduction

Gliomas, constitute a significant proportion of primary malignant brain neoplasms and present a complex challenge due to their dismal prognosis but also due to their impact on patient's neurocognitive functions (Lv et al., 2022). They are presumed to originate from glial cells and infiltrate the surrounding tissues, representing the most frequent primary brain tumors in adults (Menze et al., 2015). The treatment of gliomas necessitates a multidisciplinary approach, combining maximal or supra-maximal resective surgery, radiotherapy, and chemotherapy to optimize long-term survival and enhance the patient's quality of life (Lee and Wee, 2022). The overall prognosis is significantly influenced by factors such as tumor grade, genetic alterations, and the extent of surgical resection (van den Bent et al., 2024).

Currently, the outcomes of glioma management are measured in terms of survival and the presence of any neurological deficits. However, the intricate interplay between tumor biology, treatment modalities, and individual patient characteristics gives rise to a wide spectrum of neurocognitive changes (Adams et al., 2015). The impact on cognitive domains such as memory, executive function, attention, and language can significantly impair the quality of life and functional independence of the affected individuals. The current understanding of glioma-associated cognitive impairment necessitates a comprehensive investigation into the underlying mechanisms, risk factors, and potential modifying interventions (Lukas et al., 2019).

The aim of this manuscript is to provide a comprehensive overview of the multifaceted nature of neurocognitive dysfunction in patients with glioma. We focus on the combined influence of tumor-related characteristics, treatment-associated effects, and individual patient factors. Our primary objective is to examine how glioma-associated neurocognitive dysfunction evolves and affects patients over the course of the disease. To this end, we review the neurocognitive assessment tools currently used in clinical practice and summarize evidence on preoperative cognitive impairment. In addition, we explore the impact of surgical intervention, chemotherapy, and radiotherapy on cognitive function, while also address the role of tumor location and histopathological grade in neuropsychological changes. Finally, we discuss the potential value of cognitive function as a predictor of overall clinical outcomes.

To address these objectives, we conducted a comprehensive review of the literature and current evidence on the interactions among gliomas, therapeutic approaches, and neurocognitive outcomes. This review focuses on the factors that modify patients’ neurocognitive status.

## 2 Methodology

This systematic review was conducted in accordance with the PRISMA guidelines (Page et al., 2021). The study did not involve human subjects or patient data and therefore did not require informed consent or institutional review board approval. No external funding was obtained.

### 2.1 Search strategy

A comprehensive search of PubMed, Scopus, and Web of Science was performed to identify relevant publications from database inception to August 2025. Search terms included combinations of “glioma,” “neurocognitive function,” “cognitive impairment,” “quality of life,” and “treatment outcomes.” The search was restricted to English-language articles to ensure consistency in data interpretation. The detailed search strategy is presented in Table 1, Table 2. Manual searches of key journals and scientific conference proceedings were also conducted to identify additional relevant studies.

**Table 1:** Our systematic search strategy based on the PICO criteria.

Frame	Keywords	Search	Inclusion criteria	Exclusion Criteria	Sources
**Patients**	# 1. “gliomas” OR “supratentorial gliomas” OR “glioblastoma” OR “astrocytoma” OR “oligodendroglioma” OR “brain tumor”	#1 AND #2 AND #3	RCTs” OR “observational studies” OR “case series” Published in peer-reviewed journals English language Supratentorial gliomas Adults	studies not reporting on gliomas studies on pediatric population studies on extra-axial tumors studies solely reporting on other types of CNS tumors (e.g., spinal tumors) Non-English Irrelevant title or abstract Irrelevant full text Study design other than RCTs/observational studies/case series, including editorials, reviews (included systematic reviews), letters to the Editor, meta-analyses, original studies, experimental non-human studies	Databases (PubMed, Scopus, Embase, and Web of Science) Reference list of the gathered records
**Intervention**	#2. “neuropsychological evaluation” OR “neuropsychological test” OR “cognitive battery”				
**Comparator**	Not specified				
**Outcome**	#3. “Cognitive function” OR “psychiatric symptoms” OR “behavioral changes”				
**Study design**	# 4. “RCTs” OR “observational studies” OR “case series”				
**Time**	# 4. Search period: From 1964 to August 2025				

CPGCs, clinical practice guidelines or recommendations and Consensi; PICO, patients/intervention/comparator/outcome; TBI, traumatic brain injury.

**Table 2:** Search strategy.

Data	Search string	Results
PubMed	(glioma OR glioblastoma OR astrocytoma OR oligodendroglioma) AND (“neuropsychological” OR “cognition evaluation” OR “neuropsychological test” OR “cognitive battery” OR “neurocognitive")	1491
Scopus	(TITLE-ABS-KEY(gliomas OR brain AND neoplasms OR supratentorial AND gliomas OR supratentorial AND neoplasms OR glioblastoma OR astrocytoma OR oligodendroglioma OR brain AND tumor) AND TITLE-ABS-KEY(“neuropsychological” OR “cognition evaluation” OR “neuropsychological test” OR “cognitive battery” OR"neurocognitive"))	1808
Dimensions	Title and abstract: (glioma OR glioblastoma OR astrocytoma OR oligodendroglioma) AND (“neuropsychological” OR “cognition evaluation” OR “neuropsychological test” OR “cognitive battery” OR “neurocognitive")	1334

### 2.2 Eligibility criteria

Studies were eligible for inclusion if they included adult patients diagnosed with any grade gliomas, assessed cognitive function using standardized neuropsychological or screening tests (e.g., MMSE, MoCA, ACE-III), and evaluated cognitive outcomes in relation to treatment, tumor characteristics, or disease stage. Exclusion criteria included non-English publications, studies involving pediatric populations, and those lacking standardized cognitive assessments.

### 2.3 Data extraction

Two independent authors, M.K. and A.B. screened and selected studies based on predefined inclusion and exclusion criteria. Discrepancies were resolved through consensus or consultation with the senior author (K.F). Extracted data included study design, patient demographics, tumor features, treatment modalities, neurocognitive assessment tools, and primary cognitive outcomes.

### 2.4 Data synthesis and analysis

Included studies were summarized qualitatively based on design, methodology, and outcome measures. Studies were categorized by tumor grade (low-grade vs. high-grade gliomas) and by treatment modality (surgery, radiotherapy, chemotherapy, or multimodal therapy) to enable subgroup analysis.

### 2.5 Quality assessment

Methodological quality was evaluated using the Newcastle–Ottawa Scale for observational studies (Lo et al., 2014).

## 3 Results

### 3.1 Literature search

The review initially identified 4633 records through database searching and 17 more records through citation searching. After removing all the duplicates, we reviewed 2542 for relevance of the study title, abstracts and full texts and ultimately excluded 4625 studies. The remaining 25 articles formed the basis of our systematic review. No further articles were included after searching the reference list for potentially relevant studies (Fig. 1).

**Fig. 1:**
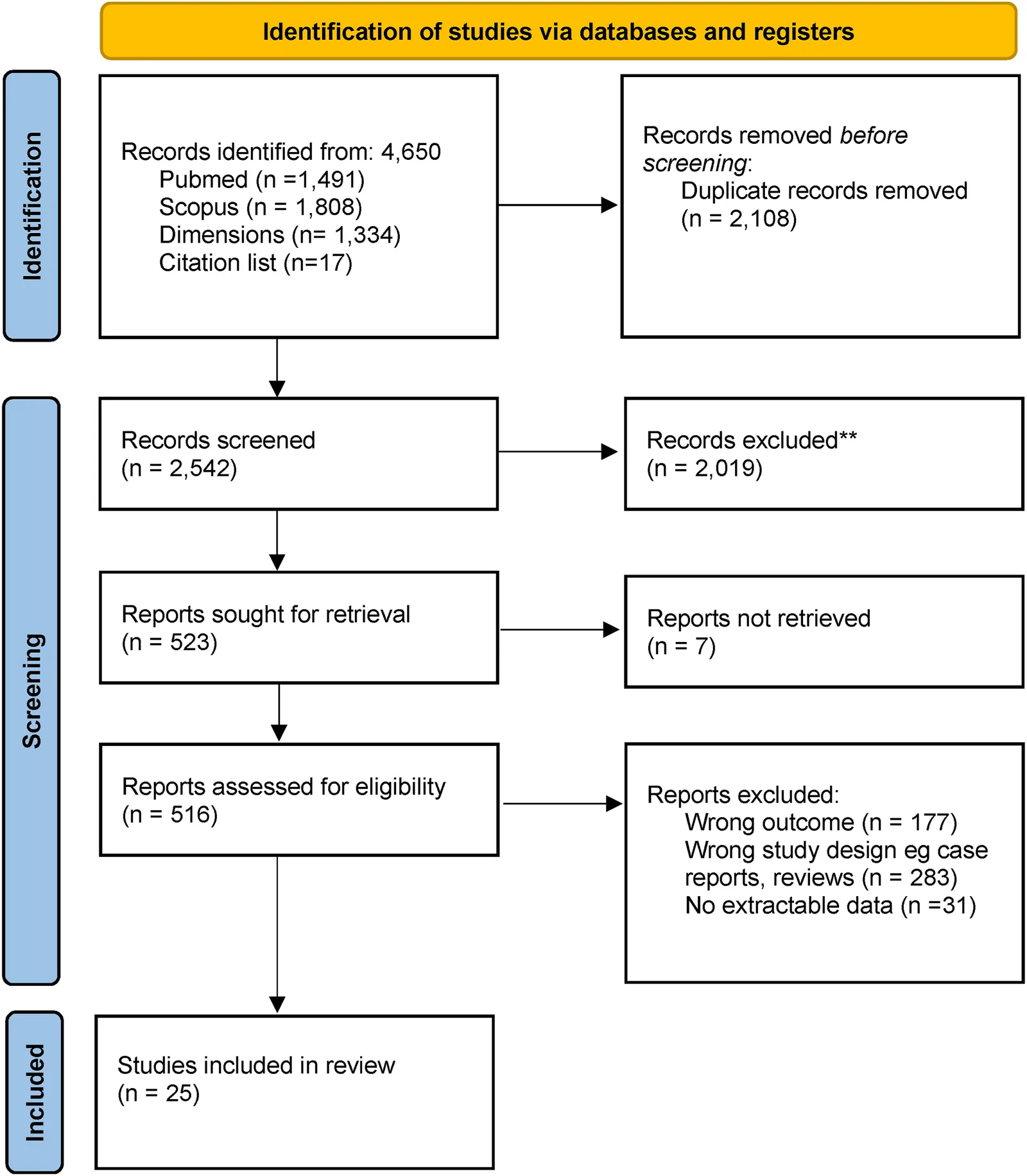
PRISMA flow diagram of our search results.

### 3.2 Study characteristics

This systematic review incorporated a total of 25 studies that met the inclusion criteria, encompassing a broad range of study types and methodologies. These included retrospective analysis (6), prospective observational cohorts (17) and case series (2). The patient population predominantly consisted of adults diagnosed with low-grade gliomas (LGG) or high-grade gliomas (HGG), the majority of whom had undergone surgical resection for their tumors. The studies varied widely in design, but all shared the common goal of investigating the neurocognitive outcomes in glioma patients, particularly in the context of surgery, treatment, and tumor location. All the eligible studies of our search are shown in Table 3. All included studies were observational cohort studies or case series. Study quality was therefore assessed using the Newcastle–Ottawa Scale (Lo et al., 2014). Based on this assessment, 10 studies were rated as having moderate methodological quality, while the remaining 15 were considered high quality (Table 4).

**Table 3:** Basic characteristics of extracted studies.

Author (Publication Year)	Study design	Patients included	Type of treatment	Tumor grade, location and laterality	Awake vs asleep craniotomy	Battery employed	Neurocognitive follow-up
Noll et al. (2016) (Noll et al., 2016)	Prospective	103	NR	Left and/or right temporal gliomas	NR	Clinical Trial Battery composite	NR
Noll et al. (2020) (Noll et al., 2020)	Prospective	15	NR	Left sided perisylvian gliomas	NR	Clinical Trial Battery composite	Pre-op Immediate post-op and 3 months post-op
Prat et al. (2020) (Prat-Acín et al., 2021)	Prospective	15	NR	Right sided LGGs	Both	Batteries for Language, spatial cognition, Social cognition, executive function, attention, working memory	Pre-op and 6 months post-op
Racine et al. (2015) (Racine et al., 2015)	Prospective	12	NR	LGGs	Awake	Batteries for short-term memory, attention, word finding, speech, reading and spelling comprehension, vision, executive function, processing speed, anxiety, Irritability, apathy, depression	Pre-op and 7 months post-op
Rinjen et al. (2020) (Rijnen et al., 2019)	Prospective	77	Surgery	Right sided or left sided LGGs	Awake and asleep	Strupp test, Verbal fluency, Shifting attention test, Verbal memory, Visual memory, Finger taping, Continuous performance, Symbol digit coding	Pre-op and 3 months post-op
Pertz et al. (2013) (Pertz et al., 2013)	Prospective	71	Surgery, chemotherapy and radiotherapy	NR	NR	Neurocog FX (battery) Domains: psychomotor speed and inhibition Verbal short term memory Working memory Verbal fluency Verbal and non-verbal memory	NR
Ng et al. (2020) (Ng et al., 2020)	Retrospective	47	Surgery	LGG	Awake	DO80 naming task and comprehensive neuropsychological battery covering multiple cognitive domains	Pre-op and 3 months post-op
Satoer et al. (2013) (Satoer et al., 2014)	Prospective	45	Surgery, chemotherapy and radiotherapy	LGG or HGG	Asleep	Language (Boston naming test, letter fluency) and batteries for memory, attention, executive function	Pre-op, 3 months post-op and 12 months post-op
Schei et al. (2022) (Schei et al., 2022)	Prospective	237	No treatment	Gliomas	NR	QLQ-C30	NR (evaluation was only pre-operatively)
Talacchi et al. (2011) (Talacchi et al., 2011)	Prospective	29	Surgery	Frontal, temporal and parietoccipital gliomas	NR	Edinburgh Handedness Inventory (EHI) Raven Coloured Matrix (Raven 47) Word fluency (FAS) Trail Making Test (TMT-A e B) Verbal Digit Span (Span) 15 Rey-Osterrieth Word List, immediate recall (ROWL, IR) 15 Rey-Osterrieth Word List, delayed recall (ROWL, DR) Spatial Supraspan Learning (TAS) Rey-Osterrieth Complex Figure (ROCF) Visual Object Naming (VON) Copy design Limb praxis Orofacial praxis Body Part Denomination (BPD) Finger Denomination (FD)	3-7 days post op
Tymowski et al. (2018) (Tymowski et al., 2018)	Prospective	21	Surgery	Right or left sided secondary motor cortex LGGs	NR	Assessment of dynamic and pose praxis according to Luria's classification Mini Mental State Examination (MMSE) Montreal Cognitive Assessment (MoCA) Frontal Assessment Battery (FAB) Attempts to name objects from Addenbrook's Cognitive Examination-III (ACE-III) Phonetic fluency tests Semantic fluency tests Rey's 15-word test (RAVLT) Diagnostic Test for Brain Damage by Hillers (DCS) Wisconsin Card Sorting Test (WCST) Ruff Figural Fluency Test (RFFT) Trail Making Test Bell test Test for visual search effectiveness	9-27 days post-op
Van Kessel et al. (2019) (van Kessel et al., 2020)	Retrospective	168	Surgery, chemotherapy and radiotherapy	LGGs and HGGs	Awake	NR	Pre-op, 3 months and 6 months post-op
Wager et al. (2012) (Wager et al., 2013)	Rerospective	9	Surgery, chemotherapy and radiotherapy	Frontal right and left sided LGGs and HGG	Awake	Stroop, WCST, TMT, WAIS, WMS	Pre-op, 3 months and 6 months post op
Wu et al. (2011) (Wu et al., 2011)	Prospective	33	Surgery, chemotherapy and radiotherapy	Right and left insular grade II and III gliomas	Awake	WAIS-III Span WAIS-III Symbol Trail-Making test, Part A Trail-Making test, Part B WAIS-III Similarities Controlled Oral Word Association Hopkins Verbal Learning Test-Revised Boston Naming Test or MAE Visual Naming MAE Token Test WAIS-III Block Design Grip Strength Grooved Pegboard	NR
Yoshi et al. (2008) (Yoshii et al., 2008)	Prospective	83	Surgery	Right or left sided LGG, anaplastic glioma and HGG	NR	3MS (Modified Mini-Mental State Examination)	Pre-op or 1 month within post operative period
Butterbrod et al. (2020) (Butterbrod et al., 2020)	Retrospective	114	Surgery and chemotherapy	HGG	NR	CNS Vital Signs, Stroop I & III	Single evaluation at 3 months post-op
Dallabona et al. (2017) (Dallabona et al., 2017)	Prospective	30	Surgery	Right and left sided HGG	NR	Extensive neuropsychological battery (34 tests across language, memory, attention, EF, praxis)	Pre-op, 7 days post-op and ∼40 days post-op
Flechl et al. (2012) (Flechl et al., 2012)	Retrospective	17	Surgery, chemotherapy and radiotherapy	HGG	NR	NeuroCogFX + QoL (EORTC QLQ-C30/BN20), HADS, motor tests	Follow-up ≥3 years after diagnosis
Habets et al. (2014) (Habets et al., 2014)	Prospective	62	Surgery	Right and left sided HGGs	NR	Neuropsychological testing across 7 domains (memory, attention, EF, psychomotor speed, etc.)	Pre-op and median ∼5 weeks post-op
Hoffemann et al. (2016) (Hoffermann et al., 2017)	Retrospective	196	Surgery	Right and left sided brain tumors	NR	NeuroCogFX computerized screening (8 subtests; overall score)	pre-op and 3–4 months post-op
Huang et al. (2014) (Huang et al., 2014)	Retrospective	12	Surgery	Frontal lobe LGGs	NR	Montreal Cognitive Assessment (MoCA) + resting-state fMRI network metrics	Pre-op and post-op (interval not specified)
Lemaitre et al. (2022) (Lemaitre et al., 2022)	Prospective	157	Surgery	Right or left sided supratentorial LGGs	Awake	Comprehensive neuropsychological battery (executive, attention, language, memory, visuospatial, social cognition)	Pre-op and 3 months post-op
Motomura et al. (2018) (Motomura et al., 2018)	Prospective	5	Surgery	Right or left sided frontal lobe LGGs	Awake	WAIS-III, WMS-R, FAB, SLTA	Pre-op and post-op (timing not specified)
Noll et al. (2015) (Noll et al., 2015)	Prospective	64	Surgery, chemotherapy and radiotherapy	Right or left sided temporal lobe LGGs and HGGs	Awake and asleep (mixed)	Comprehensive neuropsychological battery (HVLT-R, TMT A/B, COWA, Token Test, Naming, etc.)	Pre-op and 3 months or earlier post-op
Barberis et al. (2021) (Barberis et al., 2021)	Retrospective	20	Surgery	Right frontal lobe LGGs and HGGs	Awake	Extensive NP battery (DO80, TMT, Stroop, Rey Figure, Digit Span, PASAT, PPTT, 'Reading the Mind in the Eyes')	Pre-op, 1 week or immediately post-op and 4 months post-op

3MS, Modified Mini-Mental State Examination; ACE-III, Addenbrooke's Cognitive Examination, Third Edition; BN20, Brain Neoplasm Module (EORTC QLQ-BN20); BPD, Body Part Denomination; CNS, Central Nervous System; COWA/COWAT, Controlled Oral Word Association (Test); DCS, Diagnostic Test for Brain Damage (Hillers); DO80, Dénomination d’Objets 80 (80-item picture naming test); EHI, Edinburgh Handedness Inventory; EF, Executive Function(s); EORTC QLQ-C30, European Organisation for Research and Treatment of Cancer Quality of Life Questionnaire-Core 30; FAB, Frontal Assessment Battery; FD, Finger Denomination; fMRI, Functional Magnetic Resonance Imaging; FAS, phonemic verbal fluency task (letters F–A–S); HADS, Hospital Anxiety and Depression Scale; HGG, High-Grade Glioma; HVLT-R, Hopkins Verbal Learning Test-Revised; LGG, Low-Grade Glioma; MAE, Multilingual Aphasia Examination; MMSE, Mini-Mental State Examination; MoCA, Montreal Cognitive Assessment; NR, Not Reported; NP, Neuropsychological; PASAT, Paced Auditory Serial Addition Test; PPTT, Pyramids and Palm Trees Test; QLQ, Quality of Life Questionnaire; QoL, Quality of Life; RAVLT, Rey Auditory Verbal Learning Test; ROCF, Rey–Osterrieth Complex Figure; ROWL (IR/DR), Rey–Osterrieth Word List (Immediate Recall/Delayed Recall); RFFT, Ruff Figural Fluency Test; SLTA, Standard Language Test of Aphasia; TAS, Spatial Supraspan Learning Test; TMT (A/B), Trail Making Test, Parts A and B; VON, Visual Object Naming; WAIS-III, Wechsler Adult Intelligence Scale, Third Edition; WCST, Wisconsin Card Sorting Test; WMS/WMS-R, Wechsler Memory Scale/Revised.

**Table 4:** Newcastle–ottawa scale (NOS) scoring.

Author (Year)	Selection (0–4)	Comparability (0–2)	Outcome (0–3)	Total (0–9)
Noll et al. (2016)	3	1	2	**6**
Noll et al. (2020)	3	1	3	**7**
Prat et al. (2020)	4	2	3	**9**
Racine et al. (2015)	3	1	3	**7**
Rijnen et al. (2020)	4	2	3	**9**
Pertz et al. (2013)	3	1	2	**6**
Ng et al. (2020)	3	1	3	**7**
Satoer et al. (2013)	4	2	3	**9**
Schei et al. (2022)	3	1	1	**5**
Talacchi et al. (2011)	3	1	2	**6**
Tymowski et al. (2018)	3	1	2	**6**
Van Kessel et al. (2019)	4	2	3	**9**
Wager et al. (2012)	3	1	2	**6**
Wu et al. (2011)	4	2	2	**8**
Yoshii et al. (2008)	3	1	2	**6**
Butterbrod et al. (2020)	3	2	2	**7**
Dallabona et al. (2017)	4	2	3	**9**
Flechl et al. (2012)	3	1	2	**6**
Habets et al. (2014)	4	2	3	**9**
Hoffermann et al. (2016)	3	1	3	**7**
Huang et al. (2014)	3	1	2	**6**
Lemaitre et al. (2022)	4	2	3	**9**
Motomura et al. (2018)	2	1	1	**4**
Noll et al. (2015)	4	2	3	**9**
Barberis et al. (2021)	4	2	3	**9**

### 3.3 Neurocognitive assessment tools

Across the included studies, a wide array of standardized neurocognitive tests was utilized to assess various aspects of cognitive function. Some of the most frequently used assessment tools (Table 5) were:

• **General Cognitive Screening Tests**: These included the Mini-Mental State Examination (MMSE), Montreal Cognitive Assessment (MoCA), and Addenbrooke's Cognitive Examination (ACE-III), which were used to assess global cognitive function.
• **Frontal and Executive Function Tests**: Tests such as the Frontal Assessment Battery (FAB), Stroop Test, Trail Making Test (TMT), and the Wisconsin Card Sorting Test (WCST) were commonly employed to evaluate executive function, which is often affected in glioma patients, particularly in those with tumors in the frontal lobe.
• **Memory and Learning Assessments**: The Rey Auditory Verbal Learning Test (RAVLT), Wechsler Memory Scale (WMS), and Rey-Osterrieth Complex Figure Test (ROCF) were commonly used to assess various aspects of memory, including verbal and visual memory, as well as learning ability.
• **Psychomotor and Visuospatial Measures**: Tools such as the Line Bisection Test, Bells' Test, Clock Drawing Test, as well as tests from CNS Vital Signs and NeuroCog FX, were used to evaluate psychomotor speed and visuospatial abilities.
• **Social and Emotional Cognition**: The Read the Mind in the Eyes Test and Facial Emotion Recognition tasks were used to assess social cognition, an often-overlooked domain in neurocognitive assessments, but one that may be affected by tumor location, particularly in the temporal lobes.

**Table 5:** Summary of the employed neuropsychological batteries and the examined cognitive functions.

Battery	Tested Cognitive Functions
Mini-Mental State Examination (MMSE), Montreal Cognitive Assessment (MoCA), Addenbrooke's Cognitive Examination (ACE-III)	Global cognitive functions
Frontal Assessment Battery (FAB), Stroop Test, Trail Making Test (TMT), Wisconsin Card Sorting Test (WCST)	Executive thinking functions
Rey Auditory Verbal Learning Test (RAVLT), Wechsler Memory Scale (WMS), Rey-Osterrieth Complex Figure Test (ROCF)	Verbal and/or visual memory, learning ability
Line Bisection Test, Bells' Test, Clock Drawing Test, CNS Vital Signs, NeuroCog FX	Psychomotor speed, visuospatial abilities
Read the Mind in the Eyes Test, Facial Emotion Recognition	Emotional & social functions

### 3.4 Neurocognitive outcomes

The analysis of the studies revealed several consistent findings related to cognitive outcomes in glioma patients. These outcomes included pre- and post-operative findings.

### 3.5 Preoperative cognitive impairment

Cognitive impairments were commonly observed even before surgical treatment in patients with gliomas, particularly in those with tumors located in eloquent brain regions, such as the frontal and temporal lobes. These regions are responsible for higher-order cognitive functions such as executive function, memory, and language, making them highly vulnerable to tumor-related damage. There are many studies reporting that more than 40% of the glioma patients have cognitive impairments pre-operatively (Noll et al., 2016; Rijnen et al., 2019; Satoer et al., 2014; Schei et al., 2022; Dallabona et al., 2017; Habets et al., 2014; Lemaitre et al., 2022; Barberis et al., 2021). Specifically, Habets et al. (2014), reported that 79% of patients with HGG had cognitive impairment in at least one domain, while Noll et al. (2016) reported in their study, that 75% of patients with gliomas located in left and right temporal lobe had pre-operative neurocognitive impairment. Right-sided tumors affected mainly verbal memory and executive function and left-sided tumors attention and object naming (Fig. 2). Interestingly, seizures pre-operatively didn't affect patient's neurocognition (Noll et al., 2016). Rinjen et al. (Rijnen et al., 2019), also reported pre-operative deficits in all their patients, with right-sided tumors affecting cognition even more. On the other hand, Dallabona et al. (2017), identified in their article that preoperative performances in language and verbal memory tasks depended on the joint effect of tumor volume, volume of surrounding edema, and tumor localization, with major deficits in patients with left lateralized tumor, especially insular and temporal (Fig. 3).

**Fig. 2:**
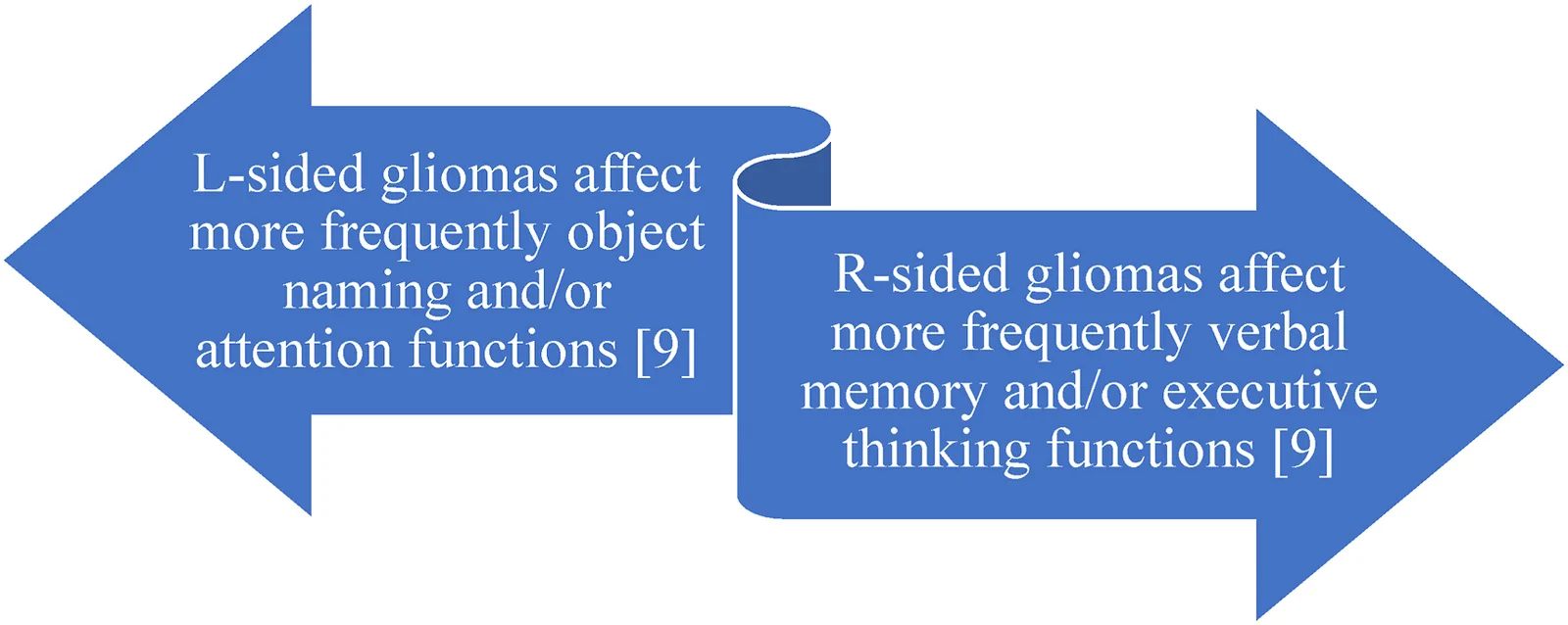
Tumor laterality has been reported to affect different cognitive functions.

**Fig. 3:**
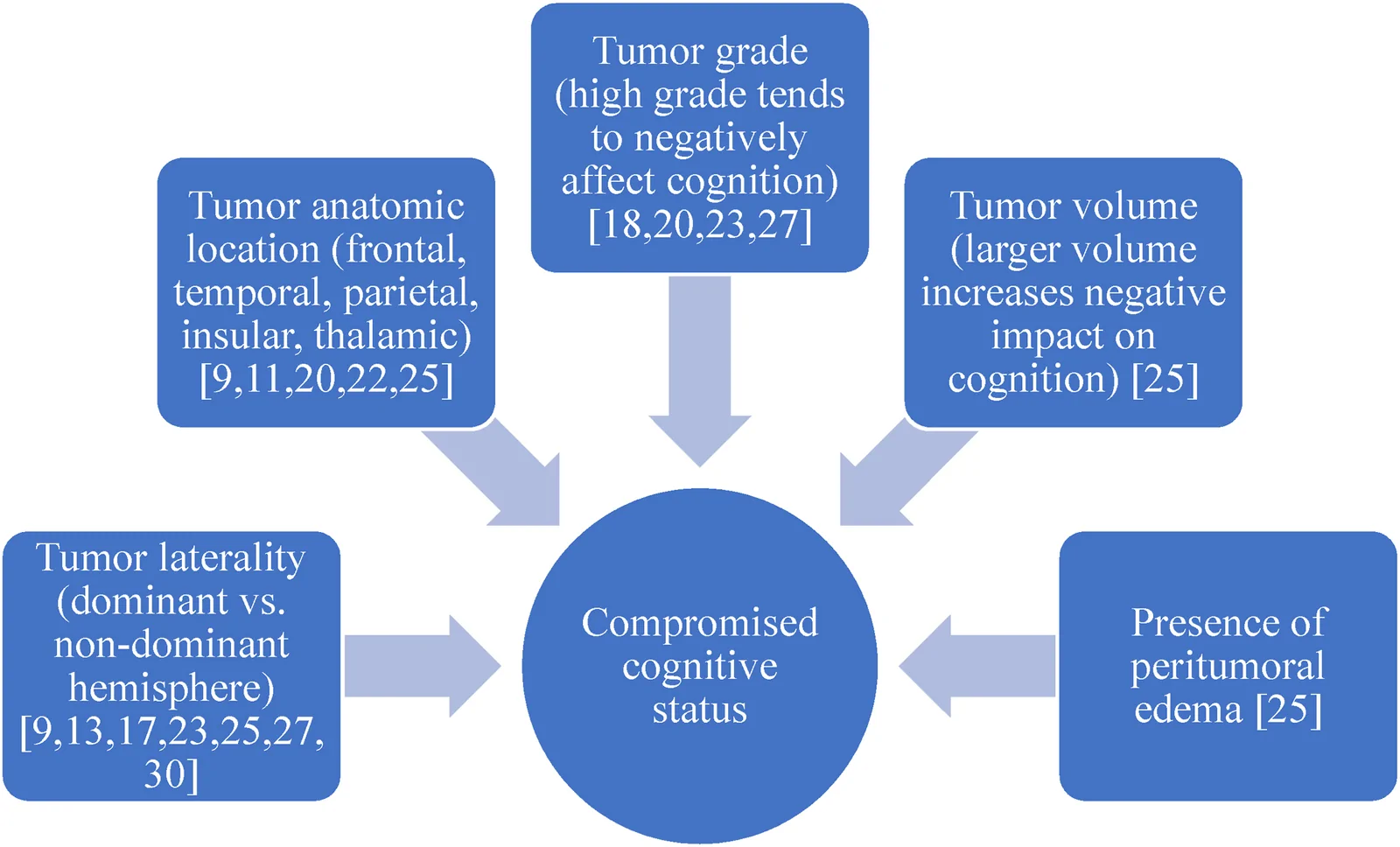
Schematic representation of reported factors affecting the cognitive status of a glioma patient.

### 3.6 Surgical impact on cognitive function

Surgery, particularly in the context of glioma resection, was often associated with a short-term decline in cognitive performance, especially in areas related to memory, executive function, and psychomotor speed. However, most studies reported that many patients experienced partial or full recovery within several months following surgery (Noll et al., 2020; Racine et al., 2015; Ng et al., 2020; Talacchi et al., 2011; van Kessel et al., 2020; Yoshii et al., 2008; Lemaitre et al., 2022; Motomura et al., 2018). This recovery, though, was not uniform, and some patients continued to experience cognitive deficits long-term, particularly those with tumors in critical areas of the brain (Noll et al., 2020; Prat-Acín et al., 2021; Wu et al., 2011; Satoer et al., 2014). In particular, Racine et al. (2015) reported that 55% of their patients had mild or transient neurocognitive deficits that improved or stayed stable in follow-up. The domain that remained mainly affected was memory. Talacchi et al. (2011), in their early assessment seven days postoperatively found low incidence of additional deficits (38%), as well as a considerable rate of early improvement. On the other hand, Sam Ng et al. (2020), reported a longer follow-up in three months postoperatively showing stable cognition in 72,3% of patients and an improvement in almost 15% of them. In their study, Van Kessel et al. (van Kessel et al., 2020) also identified that preoperative cognitive decline due to edema is associated with better postoperative results.

### 3.7 Impact of chemotherapy and radiotherapy on cognitive function

There are not many reports regarding the impact of adjuvant therapies, except surgery in cognition of glioma patients. Most of the postoperative cognitive follow-up take place before adjuvant therapies (Talacchi et al., 2011; Tymowski et al., 2018; Dallabona et al., 2017; Habets et al., 2014). However, Pertz et al. (2013) showed in their study that chemotherapy seems to affect attention, while radiation of the hippocampus affects all domains (Pertz et al., 2013). Satoer et al. (2014) and Van Kessel et al. (van Kessel et al., 2020), on the other hand, suggest that chemotherapy and radiotherapy postoperatively did not affect cognitive change (Satoer et al., 2014; van Kessel et al., 2020).

### 3.8 Location of the tumor

Location and laterality of the tumor seem to place a significant role in cognition of glioma patients. Specifically, tumors located in the left hemisphere seem to affect more cognition on glioma patients (Noll et al., 2016; Schei et al., 2022; Yoshii et al., 2008; Dallabona et al., 2017; Habets et al., 2014; Lemaitre et al., 2022) However, one study showed that right-sided tumors cause more intense worsening of neurocognition (Rijnen et al., 2019) and three more identified no correlation between tumor location and cognitive outcome. Noll et al. (2016), showed in their study that right-sided temporal gliomas affected mainly verbal memory and executive function, while left-sided temporal gliomas affected mainly attention and object-naming. Moreover, insular gliomas seem to play a significant role in cognitive worsening (Prat-Acín et al., 2021; Wu et al., 2011; Dallabona et al., 2017). Van Kessel et al. (van Kessel et al., 2020) also identified that gliomas located in thalamus have worse prognosis regarding cognition, while Dallabona et al. (2017), reported worse cognitive results in insular and temporal gliomas. Respectively, Prat et al. (Prat-Acín et al., 2021), identified that except insular gliomas, parietal gliomas also have poor cognitive prognosis.

### 3.9 Impact of awake craniotomy and intraoperative mapping

The use of awake craniotomy and intraoperative cognitive mapping techniques was frequently associated with improved preservation of cognitive function, especially for patients with tumors located in or near eloquent cortex areas (Prat-Acín et al., 2021; Racine et al., 2015; van Kessel et al., 2020; Lemaitre et al., 2022; Barberis et al., 2021). Interestingly, Rinjen et al. (Rijnen et al., 2019) is the only study that showed that patients undergoing awake surgeries had a worse cognitive outcome.

### 3.10 Differences between low-grade and high-grade gliomas

High-grade gliomas were more frequently associated with more severe and persistent cognitive impairments compared to low-grade gliomas (Talacchi et al., 2011; van Kessel et al., 2020; Yoshii et al., 2008; Habets et al., 2014). Yoshi et al. (Yoshii et al., 2008), specifically reported in their series that patients with low-grade gliomas had normal cognitive evaluation pre- and postoperative. On the other hand, Sam Ng et al. (2020) found no association between cognition and histopathology of the tumor (Fig. 3).

### 3.11 Cognitive function as a predictor of clinical outcomes

Cognitive function emerged as an important predictor of clinical outcomes in glioma patients, particularly in studies focused on long-term survivors of glioblastoma multiforme (GBM) (Butterbrod et al., 2020; Flechl et al., 2012). In these studies, cognitive performance was not only linked to overall survival but also to quality of life, highlighting the importance of preserving cognitive function as part of the overall treatment strategy (Butterbrod et al., 2020; Flechl et al., 2012).

## 4 Discussion

One of the most striking characteristics of intracranial gliomas is their impact on the neurocognitive performance of the affected patients. Although this effect was well known, the dismal prognosis of these tumors did not allow the thorough evaluation of their neuropsychological consequences. However, during the last decade several clinical series have been published evaluating the neuropsychological effects of gliomas. These publications demonstrate a wide heterogeneity evaluating different aspects of the neuropsychological profile, at different temporal phases before any treatment and/or after treatment, with various psychological batteries. They have also attempted to identify the role of each of the administered treatment (surgical resection, adjuvant radiotherapy and chemotherapy) on the observed neuropsychological changes. All these efforts, despite their high diversity, undoubtedly shed some light on this unexplored scientific landscape, and establish a platform for further research and development on this highly important clinical field.

The infiltrative character of gliomas and their fast expansion, along with their frequently associated mass effect and the development of cytotoxic edema, influence the neuropsychological profile of the affected patients frequently before even establishing their diagnosis (Zwinkels et al., 2016; Taylor et al., 1998a). The vast majority of the previously published series documented neurocognitive (memory, attention, executive function, learning deficits) and/or behavioral (anxiety, depression, irritability) changes in their patients (Racine et al., 2015; Rijnen et al., 2019; Ng et al., 2020; Satoer et al., 2014; Schei et al., 2022; Dallabona et al., 2017; Habets et al., 2014; Barberis et al., 2021). The incidence of these neurocognitive and psychological changes compared to normal, matched controls reported to be between 38 and 100% (Noll et al., 2020; Racine et al., 2015; Rijnen et al., 2019; Ng et al., 2020; Satoer et al., 2014; Schei et al., 2022). A series of factors have been identified as worsening the preoperative neuropsychological profile of glioma patients. Previous studies reported that increased tumor volume had a negative impact on the preoperative neuropsychological performance of their patients (Ng et al., 2020; Satoer et al., 2014; Schei et al., 2022). However, Noll et al. reported contradictory results with tumor volume having no effect on the observed preoperative neuropsychological deficits (Noll et al., 2015, 2016, 2020). Tumor laterality seemed to play a role in the development of preoperative neuropsychological deficits, with left-sided lesions demonstrating exacerbated cognitive deficits (Noll et al., 2015, 2016, 2020; Rijnen et al., 2019; Schei et al., 2022). The anatomic location of the tumor has also been implicated in the development of preoperative neurocognitive deficits and behavioral changes, with temporal, parietal and insular tumors to have higher incidence of preoperative deficits (Noll et al., 2016, 2020; Prat-Acín et al., 2021; Dallabona et al., 2017). Furthermore, advanced patient's age and poor Karnofsky Performance Status (KPS) score have been reported to be increasing the incidence of preoperative neuropsychological changes (Ng et al., 2020; Schei et al., 2022). The presence of seizures has been reported not to be associated with preoperative cognitive impairment (Noll et al., 2015, 2016, 2020), a finding that requires further exploration.

A wide variety of neurocognitive assessment tools have been utilized in the published clinical series, making thus any meaningful comparison between the reported results impossible. It has to be taken into consideration that none of the employed tools has been especially designed for evaluating the neuropsychological profile of patients with intracranial tumors. Likewise, none of the employed tests have been particularly developed for patients undergoing brain surgery and adjuvant treatments targeting the brain. Interestingly, Racine et al. found in their study that most of their patients with documented preoperative cognitive deficits had normal MoCA tests (Racine et al., 2015).

Studies on LGG have integrated a variety of neurocognitive tools to detect patients’ cognitive functioning level. LGG is a complex type of tumor, needing multifaceted tools for its assessment. The most common tools found across studies are the MoCA, MMSE, FAB, Stroop, TMT, Boston Naming Test (BNT), and several types of Fluency Assessments. Assessments that were not administered as broadly include the FAB, Stroop, TMT, BNT, and various fluency tests. It has been found that the utilized assessment tools are responsible for identifying and screening specific cognitive functions. The BNT and fluency assessments are most used for assessing language functions. The fluency test has also been found to help in the screening of executive functioning (Ng et al., 2020). Other batteries that are being administered to LGG patients for the assessment of executive functioning are the FAB and Stroop test, with the latter focusing specifically on attention and executive control.

As mentioned above, two batteries are the most common ones in the assessment of cognitive functions. The MoCA and the MMSE cover a wide variety of cognitive functions, thus acting as an umbrella over the other assessments. The differences between assessments lie in their cognitive functionality. It was identified that the MoCA is a test widely used for dementia and mild cognitive impairment (MCI) screening (Smith et al., 2007). This tool covers multiple cognitive areas, such as executive functioning, memory, attention, language, orientation, and visuospatial skills. The MMSE is a screening tool used for moderate to severe cognitive impairment (Molloy and Standish, 1997). This is its key differentiation from the MoCA test. The MMSE assesses the same functions as the MoCA, apart from executive functioning. This constitutes the reason why numerous researchers and clinicians use a combination of tools, rather than one tool that covers multiple areas. Therefore, the development of a unique tool, tailored for LGG, would be essential in the screening process. This novel assessment test needs to be brief and designed to be applicable specifically for surgery patients. Psychometricians will have the chance to administer only one neurocognitive tool, which would provide information on cognitive assessment across multiple brain regions. The development of a new tool will save time and effort in the data entry process.

The impact of the employed treatment on the neurocognitive and psychological status of the glioma patients can be difficulty evaluated, particularly of those patients with high-grade gliomas. The existent preoperative compromise of various cognitive and behavioral functions, the effect of the resection trauma, the toxic effects of the adjuvant radiotherapy and chemotherapy cannot be discriminated (Taylor et al., 1998b; Sleurs et al., 2025; Söderström et al., 2022). Therefore, all these perplexities need to be taken into consideration in the evaluation of the impact of each therapeutic modality in short- and long-term follow-up.

Many clinical series have reported immediate postoperative improvement of the neurocognitive status, especially when there is present preoperatively significant amount of swelling or mechanical mass effect caused by the tumor (Ng et al., 2020; Talacchi et al., 2011; van Kessel et al., 2020). Contrariwise, several clinical investigators found that surgical resection caused further deterioration of cognitive and behavioral functions (Noll et al., 2016; Racine et al., 2015; Rijnen et al., 2019). Satoer et al. reported that some long-term (at 12 months postoperatively) improvement of attention and executive functions, as well as in various language aspects (Satoer et al., 2014). They also postulated that any language-associated improvements occurred at least 8-12 weeks postoperatively (Satoer et al., 2014). Regarding the role of the extent of tumor resection and of the employment of intraoperative mapping via an awake craniotomy in cognitive and behavioral changes, the published results are contradictory (Prat-Acín et al., 2021; Racine et al., 2015; Rijnen et al., 2019; Ng et al., 2020; van Kessel et al., 2020; Lemaitre et al., 2022; Barberis et al., 2021). Although many series identified that intraoperative mapping may minimize neuropsychological negative impact, Rijnen et al. reported worse neuropsychological outcome in cases that mapping was employed (Rijnen et al., 2019).

The toxic effects of radiation therapy and chemotherapy in adult glioma cases have been adequately described in the pertinent literature. (Taylor et al., 1998b; Sleurs et al., 2025; Söderström et al., 2022). Likewise, Pertz et al. found that chemotherapy affected minimally selective attention in their patients, while radiation of the hippocampus affected all cognitive functions (Pertz et al., 2013). It should be mentioned, however, that there are published series that chemotherapy and radiotherapy had no impact on the neuropsychological profile of glioma patients (Satoer et al., 2014; van Kessel et al., 2020).

Follow-up of glioma patients for evaluating mid- or long-term neurocognitive and behavioral effects is quite challenging. Poor survival among high-grade glioma patients makes impossible the evolution of cognitive and psychological deficits over time, making thus the follow-up of younger patients with low grade gliomas more meaningful. Postoperative neuropsychological evaluation immediately before the administration of any adjuvant therapies, and thereafter every three months until the completion of a five-year period in these cases. Previous publications have demonstrated long-term improvement in several aspects of neuropsychological profile of patients with low grade gliomas (Barzilai et al., 2018; McAleer and Brown, 2015; Herbet et al., 2021).

## 5 Strengths and limitations

### 5.1 Strengths

Our current systematic review has several strengths. Initially, it focuses on one of the most important but largely understudied outcomes of glioma care: the patient's neurocognitive functions. To be more exact, the review tries to provide a holistic overview and cupture as many domains as possible such as global cognition, executive function, memory, language, visuospatial, and social cognition, while integrating the most important outcome modifiers, including tumor-, treatment-, and patient-related factors. And all these has been achieved throught a robust and reproducible methodological approach following PRISMA guidelines, and searching multiple high-quality medical databases in a systematic and standardized manner. The use of independent reviewers for study selection and a standardized quality assessment tool (NOS) strengthens the methodological rigor. Thus, our systematic evaluation of risk factors influencing cognitive outcomes could offer valuable insights for clinical decision-making and proper patient counseling.

### 5.2 Limitations

Several limitations should be acknowledged when interpreting these findings. To start with, our systematic review resulted in 25 studies, which at the first look seems impressive, but it is a small number in relation to the known neurocognitive function domains. This results from the absence of a standardized way of neurocognitive and behavioral function reporting. Moreover, all included studies were observational cohorts or case series, which are inherently prone to confounding, selection bias, and survivor bias—particularly relevant in high-grade gliomas. For this reason, we tried to assess the methodological quality while interpreting our findings. In addition there is a significant heterogeneity among the included studies. It is more than apparent that these studies vary widely in designing, patient populations, timing of assessments, follow-up duration, and most importantly, the neurocognitive tools used. Therefore, we avoided a quantitative evidence synthesis and limited our results in a narrative format. Equally important, there are large variations in the patient follow-up. This fact limits the generalizibility of our findings to the short term and may not capture the full spectrum of long-term cognitive effets, particularly in LGG cases. Finally, restriction to publications written in English may well have excluded relevant data from studies reported in other languages and could introduce language bias.

## 6 Conclusion

It has been shown that gliomas affect various aspects of cognitive and psychological functions, and these changes may be subtle. Our systematic review highlights the importance of developing structured, specific neuropsychological assessment tools for the evaluation of patients with gliomas. These need to be focusing on those cognitive and behavioral aspects that are usually affected by gliomas and need to be brief and properly designed for patients undergoing surgery. The need of using such a unified and widely accepted neuropsychological assessment tool cannot be overemphasized. It is also of paramount importance to adopt a common preoperative and follow-up pattern, in order to have comparable results between different clinical series. These developments will make an accurate and precise neuropsychological assessment part of an integrated and efficient management for glioma patients.

## Funding

This research received no funding.

## Declaration of interests

The authors declare that they have no known competing financial interests or personal relationships that could have appeared to influence the work reported in this paper.
